# A qualitative exploration of the meaning of the term “survivor” to young women living with a history of breast cancer

**DOI:** 10.1111/ecc.12847

**Published:** 2018-04-06

**Authors:** S. Rees

**Affiliations:** ^1^ Division of Health Sciences Warwick Medical School Coventry UK

**Keywords:** breast cancer, identity, survivor, young women

## Abstract

There has been a recent increase in research considering the perceptions of the term “cancer survivor” held by individuals who have or have had cancer. This article explores the meaning of the term to young women living with a history of breast cancer. Twenty women participated in semi‐structured interviews about their experience of breast cancer. The methodology was informed by social constructionist grounded theory. Three of the women interviewed said they would use the term survivor to describe themselves, but most of the women felt it did not fit with their experiences. The accounts of those who accepted and rejected the survivor identity are explored, and subthemes in the latter are “survivor as somebody else” and “cancer's ongoing presence.” This article calls into question the basing of intervention strategies on the notion of the “cancer survivor,” and the assumption that younger women favour the survivor identity. Participants struggled with the demand to live up to the ideal of the survivor, which implied a high degree of agency where in reality, cancer was a disempowering experience. Being labelled a survivor obscured ongoing impacts of cancer on the young women's lives.

## 1 INTRODUCTION

Breast cancer is the most common cancer diagnosed in women aged 18–39 worldwide, and its incidence in this population is increasing (Merlo et al., 2012). Around 20% of cases occur in women under the age of 50 (Lawrence et al., 2011). The term “cancer survivor” was introduced by a US physician (Mullan, 1985). Mullan suggested individuals be known as survivors from diagnosis to avoid distinguishing between those with better and worse prognoses, thus defining individuals as survivors from diagnosis to the end of their life. There are other definitions of the term available. For example, the European Organisation of Research and Treatment of Cancer defines a survivor as a patient without signs of active disease (Moser & Meunier, 2014). In the USA, the survivor label has been utilised to advocate for research and better health care (King, 2006; Lerner, 2003). The term remains contested due to a lack of consensus over its exact definition and meaning (e.g. Bell and Ristovski‐Slijepcevic (2013)). To launch the UK National Cancer Survivorship Initiative (NCSI), a workshop was held where people affected by cancer voted for their preferred term; 42% voted for “living with and beyond cancer,” 36% voted for “survivorship,”, with the remainder of votes split between “life after cancer,” “cancer rehabilitation” or “none of the above” (Wetherall, Baldwin, & Frew, 2008). Despite this mixed response, the NCSI, healthcare professionals and charitable organisations continue to use the term “survivor” (Khan, Rose, & Evans, 2012).

A body of research has emerged exploring how individuals who have had cancer perceive the term survivor (e.g. Davis, Myers, Nyamathi, Lewis, and Brecht (2016); Documet, Trauth, Key, Flatt, and Jernigan (2012); Kaiser (2008); Morris, Campbell, Dwyer, Dunn, and Chambers (2011); Stephenson, Fletcher, and Schneider (2013)). This work has identified that whilst some individuals do embrace the term, many do not feel it resonates with their experience. In a recent review of 23 studies, only one European study was identified (Cheung & Delfabbro, 2016). This study found that their participants, who had many different types of cancer, had diverse responses to the survivor identity (Khan, Harrison, Rose, Ward, & Evans, 2012). No studies have looked specifically at *younger* women's perceptions; however, some differences have been identified within studies of broader age ranges. For example, Kaiser (2008) found survivorship to be less salient amongst participants over 65, but did not explicitly describe the views of young participants. Helgeson (2011) explored women's views using a questionnaire (*N* = 240, mean age 59). Higher “survivor centrality” was associated with younger age, but due to the quantitative nature of this study, it was not possible to probe any further. Thus, the picture of how age shapes perceptions of survivor identity is unclear and is not based on qualitative research with women in Europe.

This article reports on one aspect of a study which explored young women's experiences of breast cancer. The aims of this article are as follows: to investigate the meaning of the term “survivor” for the young women interviewed; to illustrate the different meaning of invoking the survivor identity versus having the label applied to oneself by others; and to discuss the paradoxes which emerge when defining all people who are living after cancer treatment as survivors.

## 2 METHODS

### 2.1 Design

This study was located within the interpretive paradigm, assuming multiple meanings rather than a single “truth” about survivorship (Bryman, 2008; Charmaz, 2014). Participants were 1–10 years post‐diagnosis and aged 18–44 at diagnosis in order to reach women for whom age may have been a salient part of their experience (Dratva et al., 2009; Kato et al., 1998). Participants were accessed through a diverse group of gatekeeper organisations. Circulated information included an invitation to contact the researcher directly. Women were then asked questions to confirm they met the inclusion criteria (Table 1). No financial/material incentive was offered to participate. A target sample size of 20 was chosen as this would provide a rich yet manageable amount of data (Creswell, 2012). The study was approved by the Humanities and Social Sciences Research Ethics Committee at the University of Warwick.

**Table 1:** Inclusion criteria

Woman diagnosed with breast cancer at the age of 18–44
Diagnosed at least twelve months previously and within the last 10 years
Completed initial treatment in the UK
Not currently receiving treatment for cancer (including secondary or metastatic cancer), other than long‐term preventative treatment such as tamoxifen

### 2.2 Data collection

The topic guide (Table 2) was developed through a literature review; this article focuses on participants’ views and responses to the survivor label. Interviews began with an open question. The word “survivor” was intentionally not used in any recruitment/participant materials, and the question “Do you use the word survivor about yourself?” was not asked until late in the interview to allow participants time to use it spontaneously.

**Table 2:** Topic guide (prompts removed)

Questions/Topics
1. Would you like to start from when you first thought something was wrong?
2. Can you talk about what life has been like since the end of treatment?
3. Can you talk about the ways you think it would have been different for you if you were diagnosed when you were older?
4. When you think about your body before treatment and now, can you talk about how you feel about it?
5. Did having breast cancer change your plans for the future?
6. Can you talk about how or if breast cancer has affected you as a woman?
7. Can you talk about if breast cancer has affected your relationship with your partner?
8. Do you use the term “breast cancer survivor” about yourself?
9. Did you feel there were any expectations from others about how you dealt with it? And how about how you are dealing with it now?
10. Did having breast cancer affect your finances?
11. Can you describe any positive aspects that have come from your experience?
Is there anything else you would like to talk about?

Interviews took place in participants’ homes or another venue of their choosing, lasting 55–100 min. Written informed consent was obtained from all participants. Sensitive issues were always approached with care to minimise distress but allow women to tell their story. Interviews were transcribed verbatim, anonymised and assigned pseudonyms. The social constructionist grounded theory method was used (Charmaz, 2014), and data were analysed concurrently with data collection, allowing for investigation of emerging themes and issues (Thornberg & Charmaz, 2013).

### 2.3 Analysis

Transcribed interviews were imported into NVivo10 and analysed using Charmaz's “systematic yet flexible” guidelines (Charmaz, 2014). After initial coding summarising “what was going on” in segments of data, codes were gathered together under umbrella codes such as “feelings about body,” to ease navigation of the hundreds of codes. The second phase of coding—focused coding—produced codes which were “more directed, selective and conceptual” (2014:57) by sorting, comparing and synthesising initial codes, and making decisions about which codes were the most significant and useful in building an interpretation of the accounts. It is possible to use grounded theory to extend a theory or build understanding of a phenomenon, without developing a theory per se (Suddaby, 2006).

## 3 RESULTS

### 3.1 Participants

The age range of the participants was 22–43 at diagnosis and 26–53 at interview (Table 3). Time since diagnosis ranged between 15 months and 9 years (average 3.5 years). Three participants were pregnant at diagnosis, all with their first child (Rees & Young, 2016). All participants had completed their initial treatment (see Table 3).

**Table 3:** Sample characteristics

Characteristic	Number (percentage of sample)	Range (mean)
Age at diagnosis		
18–24	1 (5)	22–44 (33.8)
26–30	6 (30)	
31–35	4 (20)	
36–40	4 (20)	
41–44	5 (25)	
Age at interview	–	26–53 (37)
Time since diagnosis	–	15 months–9 years (3.5 years)
Ethnicity		
White	17 (85)	
Asian	1 (5)	
Black	1 (5)	
(English‐Caribbean)	1 (5)	
Sexual identity		
Undisclosed	18 (90)	
Lesbian	2 (10)	
Disability (before diagnosis)		
No	18 (90)	
Yes	2 (10)	
Relationships		
Married	12	
Civil partnered	1	
Cohabiting	4	
Single	1	
In relationship	2	
Treatment		
Mastectomy	12	
Breast conservation surgery	8	
Chemotherapy	15	
Tamoxifen	18	
Reconstruction	9	
Preventative surgeries	5	
Recruited via		
CoppaFeel	6	
Breast Cancer Care	5	
Breast Cancer UK	4	
Support Groups	2	
Snowball	2	
National Black Women's Network	1	

### 3.2 Accepting the survivor identity

Three women's accounts indicated that they had positive reactions to the use of the term survivor in relation to themselves, and they claimed the survivor identity to different extents.

There's women who didn't survive it so yeah I'm a survivor. Sarah

If it comes to y'know bite me on the bum in ten years’ time, then it does. But until then yeah I've survived it definitely. Definitely a survivor. Beverley

Although Beverley asserts she is *“definitely”* a survivor, her quote reveals the tentative and conditional nature of being a survivor. Finally, Charlotte felt it was a positive word, but her feelings about it in relation to herself were somewhat more ambivalent:

I probably have referred to myself, maybe in the way ‘Well look if I can survive this I can survive anything’. I don't know if I would refer to myself as ‘a survivor’ exactly. I'm not sure. Charlotte

Charlotte's cancer occurred in the context of her disability: she had been a wheelchair user all her life and her parents were told when she was born that she would not survive longer than 6 months.

You would expect a person with a disability to be quite fragile really, and then when you get a life‐threatening illness as well, but…I keep coming on out through the other side. Charlotte

Charlotte was speaking from the position of a young disabled woman of whom, as she said, people have certain expectations, illustrating how women draw on their biographical and experiential knowledge when configuring their identity in relation to the survivor identity.

### 3.3 Rejecting the survivor identity

The majority (*N* = 17) of the 20 young women in the study rejected the survivor identity. When the issue of the meaning of the “breast cancer survivor” was raised, the responses of women were negative, often quite vociferously so.

Oh God no, I hate that! Melanie

No! I hate it, I hate it. Tabitha

Two subthemes emerged in the responses of women who rejected the survivor identity: “the survivor as somebody else”; and “cancer's ongoing presence”.

#### 3.3.1 Survivor as somebody else

A number of the young women felt that they did not live up to the image of the survivor because it implied a sense of control which they did not recognise in their own experience.

I haven't done anything personally to enable me to survive, it's the medication and the hard work of the doctors that have kept me alive. Gemma

I had no control over it, I had to hope that other people got rid of it for me. Joanna

Women felt that the term survivor suggested that they had affected the outcome of treatment, when in reality, cancer treatment resulted in a loss of control and agency over their lives and bodies.

Others resisted the label because it made them feel as though they had come under attack, and this did not fit with their experiences.

Basically my oestrogen had a rave party in my boob…So it's not like I'm a victim of my body; it just happened. Naomi

I just feel we all have ups and downs…I don't want to hear about being a survivor, I don't want to hear about my battle with cancer, no, all that kind of language. Tabitha

The experience of being labelled, even with an apparently empowering label, can actually be disempowering and deprive an individual of a sense of agency. In the first instance, the young women felt that survivor implied they had more control, or a *higher* degree of agency, than they did in reality, whereas in the latter, the young women suggested that the label *removed* some of their agency and positioned them as victims of an attack. This highlights the diverse and complex meanings which can be ascribed to a particular term.

Some of the women rejected the term in relation to themselves, simultaneously positioning other women as survivors. For example, a number of the young women interviewed felt that they did not “qualify” as survivors.

To me it feels like because I had quite a small tumour and I didn't have chemotherapy, and yeah I'm on medication [but] it's not as serious as the people who've had **serious** breast cancer. Philippa [participant's emphasis]

When they're given a certain limiting time [and] they surpass all that and y'know they've got…the strength of body and mind to get through it. Joanna

Almost all of the women who drew on this narrative did not receive chemotherapy as part of their treatment, and they appeared to consider women who had chemotherapy as closer to a terminal diagnosis.

I don't feel like I was near death or anything. Ailsa

I feel like I had a really close brush with something. Evelyn

Although they felt that the word survivor did not apply to them, they accepted it in relation to other women, such as those who had more invasive and unpleasant treatment (i.e. chemotherapy), or who outlived their prognosis. This suggests a hierarchy perceived by those who have been treated for breast cancer, with those given chemotherapy at the top. Chemotherapy is of course an extremely toxic treatment which also results in visible effects such as hair loss, a social signifier of cancer (Mathieson & Barrie, 1998). However, there was another woman who did have chemotherapy but who drew on the theme of not being close enough to death:

I don't feel like I've survived anything because it was caught so early that it, that's what medical treatment is there for: get rid of it, move on. Catherine

Catherine's mother had been previously successfully treated for breast cancer, and this added to her confidence that she would not die from it.

Most of the young women rejected the survivor label, sometimes vehemently, sometimes suggesting that older women were the survivors.

Naomi: “Maybe for like older women it's more of an appropriate [label]…it just makes it sound like a bit of a club and for some people that's good”

Interviewer: “You don't feel part of that club?”

Naomi: “No, I'm too young. If I was say fifty…”

When you're young it's just a bit of a pathetic word…I feel they're another generation in a way so it's quite different to what I'm going through. Melanie

When the young women recounted their experiences of diagnosis and treatment, they often described feeling out of place in clinics and support groups. The sense that this should not be happening because they were too young was reinforced by in health settings.

Every time you went to a new department for a different kind of scan or saw a different nurse for like a blood test, they were like ‘Oh you're so young!’ Like **every** time…It was like you were some alien. Faith [participant's emphasis]

It does feel as though it's an older woman's condition…They are all silver‐haired Goddesses sitting in that waiting room…People think you're there for someone else. Philippa

For similar reasons, mainstream support groups were found to be unhelpful and even distressing to some of the young women.

They were in such a different place in their life to me…I just left at one point just to have a cry in the toilets. Joanna

Young women with breast cancer may feel alienated from mainstream breast cancer “survivor culture,” perceiving older women to belong to that “club” and not them. They felt that their circumstances were so different to older women that they could not have anything in common.

#### 3.3.2 Cancer's ongoing presence

A major subtheme in the responses of women who rejected the survivor identity was the ongoing presence of cancer in their everyday lives. For many of the young women, there was profound uncertainty about whether they were truly free of cancer, even those living 9 years beyond treatment.

You just don't ever get the all clear. Yeah I don't think I'm ever gonna feel like a survivor. Lyndsey

I think there's a bit of a thing of not knowing if it's gone…Worrying if it's still there lurking, so…I wouldn't describe myself as a survivor. Dawn

Despite being 9 years on from treatment, Ruth was still experiencing the effects of treatment in her everyday life:

I didn't expect to have like **years** of not feeling well…The days I feel well are drowned out big time by days when I'm just not coping. Ruth

The reality of ongoing symptoms (e.g. menopausal symptoms and neuropathic pain) disrupted the expectation of a return to normality.

A malevolent power was ascribed to cancer, and young women felt that it would be “tempting fate” to say they were survivors.

I'm getting through it quietly…I don't want it to notice me and I feel like if I go ‘I'm a woman I'm a survivor!’ it'll go ‘Yeah really?’ [Laughs] And come back for a second go. Philippa

If I say ‘I've survived’ maybe the bugger will come back and bite me on the backside and say ‘Ah you've not bloody survived now have you!’ Ruth

The ongoing fear of recurrence had particular meaning for them as younger women, as their perceptions about the future were altered, and fears about recurrence were magnified by the possibility of so many decades ahead during which breast cancer could recur.

If I get it back when I'm 40, which might not happen but there's still a huge chance that it will, I'm still like super young really at 40…It will just taint my whole future, I know that I'll never be able to stop thinking about it, worrying about it. Lyndsey

Other women spoke candidly about the losses they experienced as a result of cancer. For example, Vanisha's sister died from breast cancer during Vanisha's own treatment:

It isn't something that's been and gone for me…It's still with me on a physical level, it's still with me on a psychological level, it's still with me at the level of the losses that I've experienced both in terms of my own body, my sister. Vanisha

In contrast to these experiences, the young women found others around them expected them to be well and restored to some kind of normality.

You don't want to keep whingeing about it and you don't want to keep bringing it up and it's like ‘Yeah get over it Lyndsey’ [Laughs] ‘You're like a year on now shut up’…They wouldn't say it but I think maybe they think it. Lyndsey

I hide it massively, like I can put on such a good front…’Oh she's so inspirational’…But they don't know the rest of the crap I don't tell people. Melanie

Not only were the women experiencing profound disruption to their lives and suffering with deep uncertainties, but they felt unable to communicate these to some key others because of expectations imposed upon the young women about cancer survivorship. This left them feeling isolated and disempowered.

Everybody thinks I'm back to normal it's kind of like ‘Oh you're fine now, you beat it’. I haven't beat it I'm still, not even in remission they don't even call it that it's kind of like I'm still trying to prevent it from coming back I could still, it could still come back and y'know and you kind of want to shake people and go ‘I'm not okay, just listen to me!’ Gemma

Their apparent return to “normal” meant that emotions perceived to be negative, such as worry or fear, were not expected now that they had moved into the survivor role, and the women felt restricted in which emotions they could express.

## 4 DISCUSSION

Interviewing young women about their perspectives on the “breast cancer survivor” identity revealed much about our shared conceptualisation of cancer and life beyond. A number offered a strong critique of the concept, surprising given that most were recruited via and were involved in charitable organisations which use the term. These perceptions were found across the age range and time since diagnosis.

For three women, the term was a source of pride, accurately described their experience or made sense in the wider context of their lives, but most of the young women felt it did not resonate. Previous research suggested that younger age was associated with stronger association with the survivor identity, but this study contradicts this. Research with women over 70 suggests that the label does not resonate with them because they perceive cancer to be of less importance within the context of other life experiences or health issues (Khan, Harrison, et al., 2012; Pieters & Heilemann, 2011). Age may well be a factor, but perhaps the label is most embraced by women aged for example over 45 but under 70. Further research could explore this.

As in previous research (Kaiser, 2008; Khan, Harrison, et al., 2012; Pieters & Heilemann, 2011; Trusson, Pilnick, & Roy, 2016), a number of the young women felt they had not come close enough to death to be considered survivors. There appeared to be a hierarchy perceived by some women living beyond breast cancer, and women who did not have chemotherapy felt undeserving of the survivor accolade. Cancer treatment can result in the loss of a sense of agency or control of one's body (Dunn & Steginga, 2000; Little, Jordens, Paul, Montgomery, & Philipson, 1998; Thomas‐MacLean, 2004). Some participants felt they did not deserve to be labelled survivors because it implied they did something proactive to survive. These responses highlight the paradox of referring to a person living beyond cancer as a survivor because it suggests a sense of control and purposeful overcoming.

The participants felt vulnerable given the many decades ahead of them during which cancer could recur, and they also felt that if it did recur, it would at a time when they would still be “too young” for cancer. This echoes findings from Hesse‐Biber's study who found that women who had a family history of cancer perceived a “cancer clock” (Hesse‐Biber, 2014), or a timeline when they thought they were likely to get cancer, based on the age at which their relatives had been diagnosed. Similarly, the young women in this study took into account not only clinical and scientific knowledge about risk, but also their own experiential and biographical knowledge when they considered whether they were cancer survivors.

The young women described how the reality of living beyond breast cancer treatment challenged the expectations of survivorship. They felt unable to express their ongoing fears and symptoms, as people expected them to return to normality. The survivor is cast as “someone made normal” by others (Little, Paul, Jordens, & Sayers, 2002), leaving little space for expressing ongoing effects of treatment or fear of recurrence, and women felt confined by these expectations. All three of the women who said that they would refer to themselves as survivors experienced profound uncertainty (Rees, 2017), and cancer as an ongoing presence, suggesting that it *is* possible to embrace the survivor identity whilst living with the uncertainty surrounding it.

The young women's responses drew attention to the issue of agency (Archer, 1995). The three women who embraced the survivor identity did so by actively invoking it in relation to themselves on their own terms, rather than having it imposed on them by others. However, the other women in the study resisted having the label, or any label in relation to breast cancer, imposed upon them by others. They demonstrated their agency by rejecting it, but they also highlighted the limits to this, and the impact of being labelled in a way which makes one uncomfortable. Both the adoption and rejection of a survivor identity are valid and can be understood as a way of regaining one's agency after the disempowering experience of cancer diagnosis and treatment.

### 4.1 Limitations and implications for further research

The sample size inevitably restricts generalisability of the findings. However, small qualitative studies such as the present research could inform the design of a larger study. Literature about black and minority ethnic, sexual minority and disabled women in the field of breast cancer is sparse but suggests that there may be particular ways that they make sense of their experience (Banning & Hafeez, 2010; Boehmer, Linde, & Freund, 2007; Davis et al., 2016; Fish, 2010; Jabson, Donatelle, & Bowen, 2011; Patel, Harcourt, Naqvi, & Rumsey, 2014; Rubin & Tanenbaum, 2011). The inclusion of five women who identified as belonging to these groups contributes their voices to the literature, but it would be wrong to suggest that such small numbers are representative of all minority women in Europe. Future research should include women from these groups in greater numbers. To demonstrate validity (Creswell, 2012; Green & Thorogood, 2013), the methods of data generation and analysis have been made explicit, supportive evidence provided for each interpretation, and deviant cases discussed. The processes described for collection and analysis enabled immersion in the data throughout the study. Further research might explore this question in other populations of young people living beyond cancer.

### 4.2 Implications for clinical practice and policy

It has been suggested that overuse of the survivor label may present a barrier to seeking help for ongoing physical and emotional issues (Little et al., 2002; McKenzie & Crouch, 2004; Pertl, Quigley, & Hevey, 2014; Stephenson et al., 2013). This research has illustrated this very point, as some of the young women in the study felt constrained by expectations of the survivor role so that they were unable to share their ongoing physical and emotional problems. Clinicians should be alert to the continuing issues people who have had cancer may face and understand that patients may not wish to raise these themselves because of the expectations upon them. Patients should be encouraged to speak up about any challenges they need help with.

## 5 CONCLUSION

This study challenges previous research which found that younger women were more likely to identify as survivors. The findings trouble the use of the term survivor in everyday and academic usage to refer to young women with a history of breast cancer. It also contributes to the growing body of literature which critiques the concept of survivorship and highlights ambivalence and discomfort with the survivor label. Health professionals and charitable organisations in the UK should take note that not all individuals living beyond cancer identify as survivors and that this language may indeed be alienating and harmful for the well‐being of many.

## CONFLICT OF INTEREST

No conflict of interests are declared by the author.
